# Multi-Omics Data Analyses Identify B7-H3 as a Novel Prognostic Biomarker and Predict Response to Immune Checkpoint Blockade in Head and Neck Squamous Cell Carcinoma

**DOI:** 10.3389/fimmu.2021.757047

**Published:** 2021-10-05

**Authors:** Wanzun Lin, Yanyan Xu, Jing Gao, Haojiong Zhang, Yun Sun, Xianxin Qiu, Qingting Huang, Lin Kong, Jiade J. Lu

**Affiliations:** ^1^ Department of Radiation Oncology, Shanghai Proton and Heavy Ion Center, Fudan University Cancer Hospital, Shanghai, China; ^2^ Shanghai Key Laboratory of Radiation Oncology (20dz2261000), Shanghai, China; ^3^ Shanghai Engineering Research Center of Proton and Heavy Ion Radiation Therapy, Shanghai, China; ^4^ Department of Gynaecology and Obstetrics, Shanghai Jiangqiao Hospital, Shanghai, China; ^5^ Department of Radiation Oncology, Shanghai Proton and Heavy Ion Center, Shanghai, China

**Keywords:** B7-H3, HNSCC, prognostic biomarker, immunotherapy, immune checkpoint

## Abstract

B7 homolog 3 (B7-H3) is a recently found superfamily B7 molecule and therefore has significant involvement in immunological regulation. However, the relationships of B7-H3 expression with the tumor microenvironment (TME), response to immunotherapy, and prognosis in head and neck squamous cell carcinoma (HNSCC) are still unknown. In the present analysis, we determined B7-H3 as a novel biomarker that predicts the prognosis and response to immunotherapy in HNSCC. B7-H3 expression is enhanced in HNSCC compared to normal sample and is stably expressed in HNSCC cell line. Besides, high B7-H3 expression is correlated with a dismal prognosis and resistance to immunotherapy and contributes to an immunosuppressive microenvironment. Moreover, single-cell RNA sequencing (scRNA-seq) analysis shows that B7-H3 is mainly expressed in the stromal as well as malignant cells. In conclusion, the study provides insight in understanding the prognostic value of B7-H3 in HNSCC and highlights its involvement in promoting the immunosuppressive microenvironment, which presents an attractive strategy for antibody-based immunotherapy.

## Introduction

HNSCC is the sixth most common malignancy globally, with a highly aggressive and heterogeneous phenotype (1, 2). Even with new targeted therapies and advancements in chemoradiotherapy, the average overall survival (OS) and progression-free survival (PFS) rates are dismal (3). Immunotherapy has now been successful in many solid tumors including HNSCC, notably immune checkpoint inhibitors which hold great promise for treating malignancies. However, only a few patients experience clinical benefits from immunotherapy (4–7). Thus, it is critical to identify a novel target for immunotherapy and exploit predictive biomarkers that stratify patients who may benefit from immunotherapy in head and neck cancer.

B7 superfamily molecules play essential roles in regulating anti-tumor immunity. For instance, B7-H1 termed as programmed death-ligand 1 (PD-L1) is a critical immune checkpoint molecule that has a predominantly inhibitory role in adaptive immunity and suppresses proliferation as well as activation of T cells (8–10). The US Food and Drug Administration (FDA) has authorized the inhibitors of the immunological checkpoint - PD-L1 for the clinical applications in HNSCC. The recently discovered B7 superfamily molecule, B7-H3, is also called CD276 (11). Unlike B7-H1 that has been extensively studied, the involvement of B7-H3 in tumor microenvironment (TME) regulation remains largely known. Previous reports have shown that B7-H3 is overexpressed in several tumor tissue types, which limit CD4+ and CD8+ T cells proliferation and may be exploited as a potential immunotherapy target (12–15).

This study aimed to (i) identify the relationship of TME and B7-H3 expression in HNSCC; (ii) evaluate its prognostic value; (iii) exploit B7-H3 as a biomarker that predicts the response of immune checkpoint therapy; (iv) elucidate the potential signaling pathways of HNSCC in the development and progression of HNSCC.

## Materials and Methods

### Cell Culture

The immortalized NP69 nasopharyngeal epithelial cell line has been bought from MeisenCTCC (cat# CTCC-004-0103, Zhejiang Meisen Cell Technology Co., Ltd, China) and was cultured in Keratinocyte medium (cat# 2101, ScienCell, USA). CAL-27 and Tca-8113 cell lines were cultured in RPMI-1640 medium (cat# 11835055, Invitrogen, USA) with 10% fetal bovine serum (cat# F8318-500ML, Sigma, USA) and 1% penicillin-streptomycin (cat# 15140155, Invitrogen, USA).

### Flow Cytometry (FCM) Analysis for B7-H3 Expression on the Cell Membrane

Briefly, cells were harvested and stained with fluorescein isothiocyanate (FITC)-anti-human B7-H3 (cat# ab275660, Abcam, UK) at 4°C for 0.5 h. After incubation, the mean fluorescence intensity (MFI) of B7-H3 was determined by a flow cytometer (CytoFLEX S, Beckman Coulter, USA).

### Data Acquisition and Processing

The gene expression dataset and associated HNSCC clinical information have been acquired from The Cancer Genome Atlas database (TCGA) (https://cancergenome.nih.gov/). The proteomics and DNA methylation were collected from the clinical proteomic tumor analysis consortium (CPTAC; http://proteomics.cancer.gov/programs/cptac) (16). IMvigor210 cohort samples who received an anti-PD-L1 agent (atezolizumab) treatment were downloaded and studied to identify the prognostic value (17). RNA-seq data, as well as therapeutical information of immunotherapy outcome, were accessed by IMvigor210CoreBiologies (http://research-pub.gene.com/IMvigor210CoreBiologies), a fully documented R statistical computing environment software and data package. The single-cell RNA-seq data were obtained from the GEO database (Smart-seq2 data, GEO: GSE103322 and GSE139324) (18, 19).

### Immune Cell Type Fractions Estimation

CIBERSORT was performed to quantify the 22 types of immune cells infiltration among each HNSCC sample (20). A leukocyte gene matrix comprising of 547 genes was used to differentiate twenty-two immune cells in the CIBERSORT system (https://cibersort.stanford.edu/) including plasma cells, memory B cells, naive B cells, T cells CD4 naïve, T cells CD8, activated CD4 memory T cells, resting CD4 memory T cells, T cells regulatory (Tregs), T cells follicular helper, monocytes, T cells gamma delta, NK cells activated, NK cells resting, macrophages: M0; M1; M2, activated dendritic cells, resting dendritic cells, resting mast cells, activated mast cells, neutrophils, & eosinophils. The relationship between immune cells infiltration and B7-H3 expression was determined using the Pearson correlation coefficient.

### Gene Set Enrichment Analysis (GSEA)

GSEA algorithm was performed to reveal the altered signaling pathways in the B7-H3 low- and high group, as recognized by their enrichment in the MSigDB Collection (h.all.v.7.4.symbols.gmt). For each analysis, gene set permutations were carried out 1000 times.

### Prediction of Response to Immunotherapy and Chemotherapy

Tumor immune dysfunction and exclusion (TIDE) analysis has been conducted to determine ICB response. Jiang et al. designed an analytic technique known as TIDE (http://tide.dfci.harvard.edu/) which enables a prediction of ICB response using two major tumor immune evasion mechanisms: T cell dysfunction induced in tumors with high cytotoxic T lymphocyte (CTL) infiltration and T cell infiltration inhibited in tumors with low CTL level (21, 22).

Data from genomics of drug sensitivity in cancer (GDSC) was used to evaluate the response to chemotherapy by performing the R package “pRRophetic” (23). GDSC is a public database that characterizes 1000 human cancer cell lines and screens them with 100s of compounds. GDSC also provides drug response data and genomic markers of sensitivity.

### The scRNA Sequencing Analysis

The tumor immune single-cell hub (TISCH) is used to analyze single-cell RNA-seq data derived from the GSE103322 and GSE139324. TISCH (“http://tisch.comp-genomics.org/home/”) is a scRNA-seq data source that focuses on TME and gives specified annotation of cell-type at a single-cell level, which allows TME exploration across multiple cancers (24).

### Statistical Analysis

The Wilcoxon signed-rank test has been applied for comparing B7-H3 expression. Receiver operating characteristic (ROC) curve was used to determine the diagnostic value of B7-H3 using R software. To assess the prognostic value, Kaplan-Meier analysis was carried out with the Survival and Survminer R package. The statistically significant P < 0.05 was applied.

## Results

### B7-H3 Is Overexpressed in HNSSC and Is Located in the Cell Membrane

**Figure 1:**
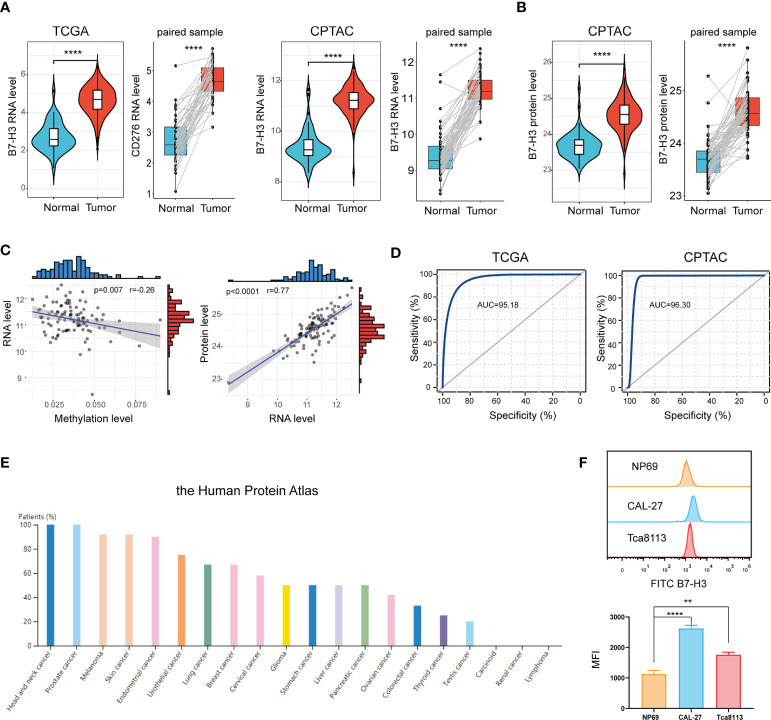
B7-H3 is overexpressed in HNSCC. **(A, B)** Violin plot and box plot present the differentially expressed B7-H3 between HNSCC and normal sample; **(C)** Scatter plots show the association of B7-H3 RNA expression with protein expression and DNA methylation; **(D)** ROC curves indicate the diagnostic value of B7-H3; **(E)** The protein expressions of B7-H3 in different tumors; **(F)** Expression level of B7-H3 in NP69, CAL-27 and Tca8113; **P < 0.01 & ****P < 0.0001.

Data from the TCGA database signified that B7-H3 is extremely elevated in tumor samples compared to normal samples or adjacent samples (**Figure 1A**). This is further validated in the CPTAC cohort. Proteins are the main executors in the biological processes. We, therefore, compared the B7-H3 protein level between tumor and normal sample. In line with the result from RNA expression, proteomics exposed that B7-H3 protein is overexpressed in the tumor sample of HNSCC (**Figure 1B**). To further unveil the underlying mechanism of B7-H3 differential expression, we examined the relationship between RNA expression and B7-H3 methylation level. The scatter plot presented a negative correlation association between B7-H3 methylation level and RNA expression (**Figure 1C**). Hence, the hypomethylation of B7-H3 DNA may be responsible for the elevated RNA expression in HNSCC. Besides, B7-H3 RNA expression showed a high positive correlation with protein expression indicating that the dysregulation of B7-H3 expression is mainly attributed to the transcriptional level rather than translational level or post-translational control (**Figure 1C**).

We generated a ROC curve to measure the diagnostic value of B7-H3 in HNSCC. The area under the ROC curve was 95.18%, with a greater diagnostic value, and CPTAC with a cohort AUC = 96.30% validated this finding further (**Figure 1D**). We further explore B7-H3 protein expression across 20 types of tumors using data from the Human Protein Atlas. Among them, HNSCC presented the highest expression level of B7-H3 with a 100% high/medium expression rate (**Figure 1E**).

To further validate the differential expression of B7-H3, we conducted a flow cytometry (FCM) analysis in HNSCC cell lines (CAL-27 and Tca8113) and immortalized NP69 nasopharyngeal epithelial cell line (**Figure 1F**). As shown in **Figure 1F**, B7-H3 is overexpressed in HNSCC cell lines (CAL-27 and Tca8113) compared to NP69.

### B7-H3 Overexpression Predicts a Dismal Prognosis and Is Associated With the Immunosuppressive Microenvironment

**Figure 2:**
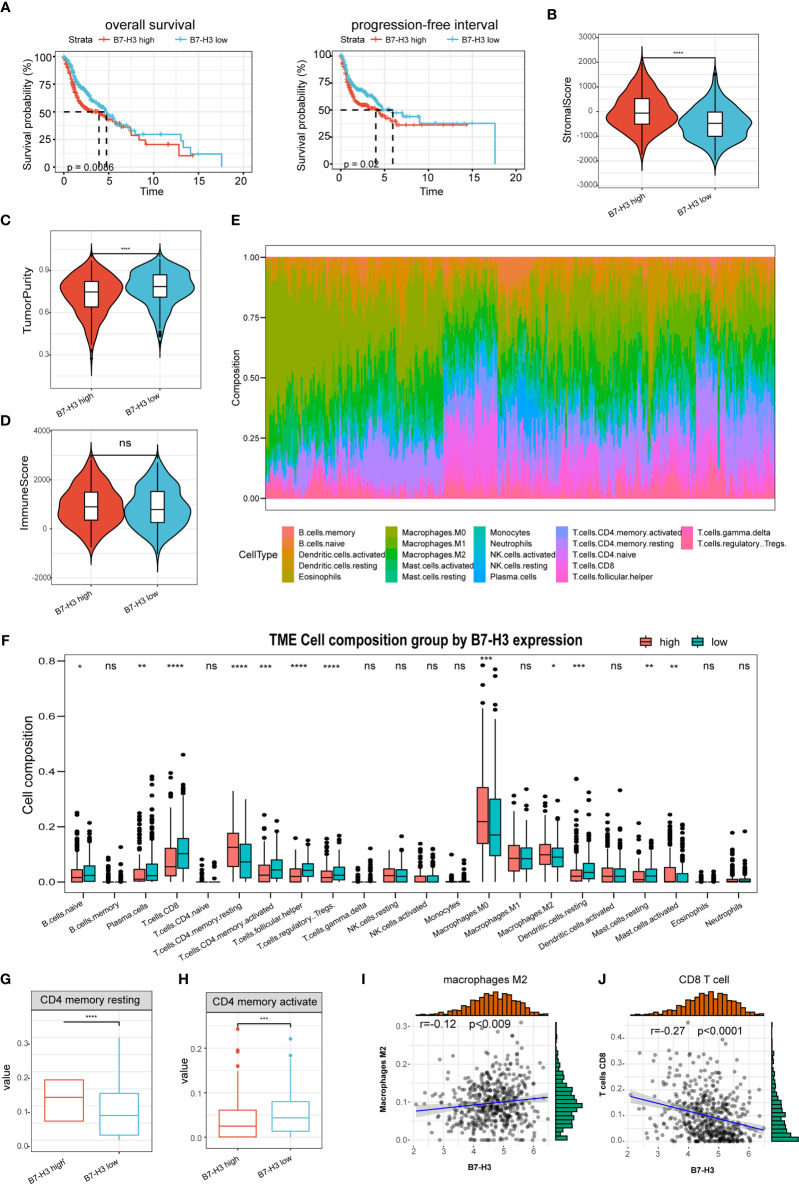
The association of B7-H3 with tumor microenvironment and prognosis. **(A)** Kaplan–Meier plots show the prognostic significance of B7-H3 in HNSCC; **(B–D)** Violin plots present the differential stromal score **(B)**, tumor purity **(C)**, and immune score **(D)** between B7-H3 low-and high-groups; **(E)** Relative proportion of immune infiltration in HNSCC patients; **(F)** box plot shows the differential immune infiltration between B7-H3 low- and high-groups; **(G, H)** Box plots visualize considerably different CD4 memory resting cells **(G)** and CD4 memory active cells between B7-H3 low-and high-groups; **(I, J)** Scatter plots show the relationship of B7-H3 expression with macrophage M2 cells **(I)** and CD8 T cells **(J)**; *P < 0.05, **P < 0.01, ***P < 0.001, & ****P < 0.0001. NS, P ≥ 0.05.

The predictive value of B7-H3 was then determined in HNSCC. Kaplan-Meier analysis discovered that the elevated B7-H3 expression is related to a short PFS and OS (**Figure 2A**). Earlier reports have exhibited that B7 superfamily molecules are critical immunomodulatory factors in the tumor microenvironment. B7-H3 is a recently found superfamily B7 molecule and therefore may have significant involvement in tumor microenvironment regulation. In our research, we show that the stromal scores were much higher in the high group of B7-H3 than in the low group of B7-H3 (**Figure 2B**). Besides, opposite trends were reported in tumor purity between the two subtypes, and no significance was observed concerning the immune score (**Figures 2C, D**).

The immune landscape of twenty-two immune cell types in HNSCC patients was shown by using the CIBERSORT method (**Figure 2E**). High B7-H3 expression patients had considerably greater proportions of immunosuppressive cells (Macrophages M2) and rested CD4 memory cells (**Figures 2F, G**), however substantially fewer proportions of the activated CD4 memory cell (**Figure 2H**). Besides, B7-H3 expression has a positive association with macrophages M2 infiltration while negative with CD8 T cell (**Figures 2I, J**).

### Validation of B7-H3 *via* scRNA-Seq

**Figure 3:**
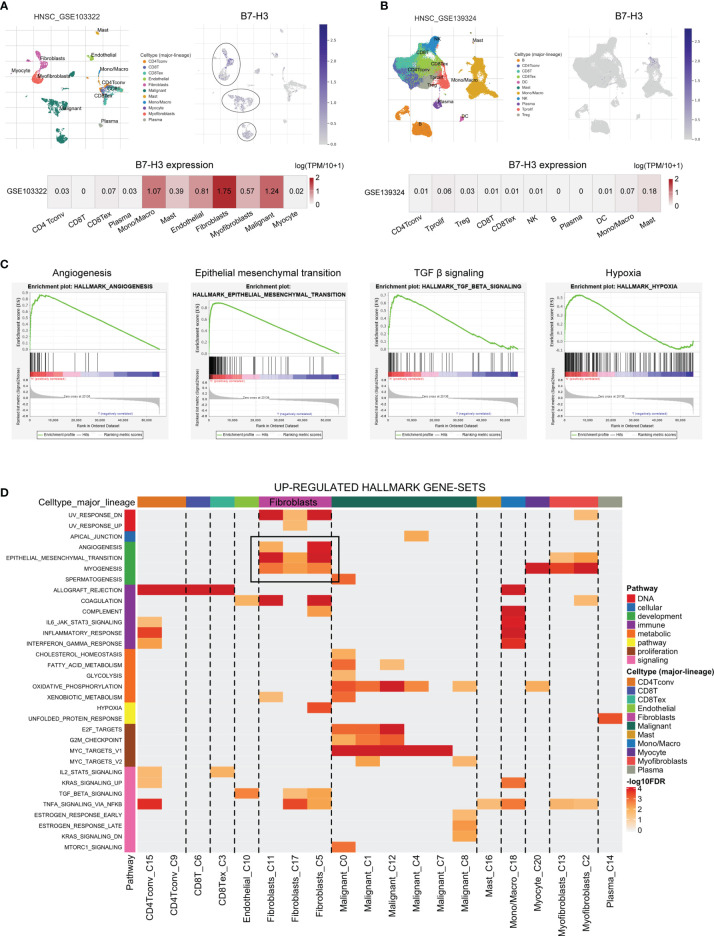
The single-cell RNA sequencing analysis exhibits the expression pattern as well as the signal pathway of B7-H3. **(A, B)** The t-SNE projection of all cells and B7-H3 expression from GSE103322 and GSE139324; **(C, D)** GSEA derived from bulk RNA-seq **(C)** and scRNA-seq **(D)** data presents the underlying pathway associated with B7-H3.

To further verify the specific type of cells that express B7-H3 in the tumor microenvironment, we conducted HNSCC scRNA-seq using data from HNSCC-GSE103322 and HNSCC-GSE139324 *via* the TISCH website (TISCH, a scRNA-seq database, offers an extensive cell-type annotation at a single-cell level, which allows TME exploration across various kinds of cancer). The result of t-distributed stochastic neighbor embedding (t-SNE) presented that 11 clusters were identified in HNSCC-GSE103322 and HNSCC-GSE139324. For HNSCC-GSE103322 data, B7-H3 was mainly expressed in the stromal cells (e.g., fibroblasts, myofibroblasts, and endothelial) and malignant cells, while B7-H3 is almost undetectable in the immune cells (**Figure 3A**). Similar results were validated in the HNSCC-GSE139324 cohort (**Figure 3B**). We conducted GSEA analysis for comparing the low- and high-expression groups in B7-H3 using TCGA-HNSCC bulk RNA-seq data to validate related signaling pathways. In the elevated B7-H3 expression groups, the gene sets were differently enriched by mechanisms that promote the cancer progression and suppress anti-tumor immunity like angiogenesis, epithelial-mesenchymal transition, TGF β signaling, and hypoxia (**Figure 3C**). Data from scRNA-seq revealed that angiogenesis and epithelial-mesenchymal transition signaling pathways were mainly enriched in fibroblasts cells (**Figure 3D**).

### Identification of B7-H3 as a Biomarker for Predicting the Response of Immunotherapy

**Figure 4:**
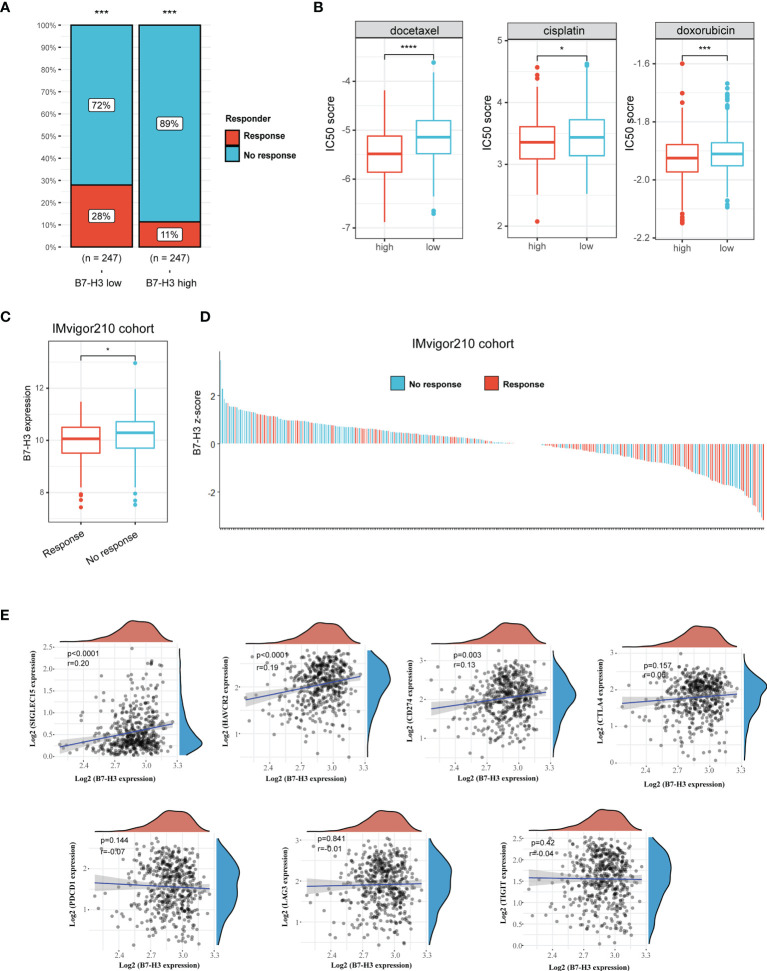
B7-H3 serves as a biomarker that predicts the response of immunotherapy. **(A)** Bar plot presents response rate of immunotherapy between high and low B7-H3 expression; **(B)** Predictive IC50 scores of docetaxel, cisplatin, and doxorubicin between high and low B7-H3 expression; **(C)** Box plot shows the differential B7-H3 expression between response and no response patients treat with ICB therapy in IMvigor210 cohort; **(D)** Waterfall plot depicts the B7-H3 expression (z-score) for a response as well as no response patients in IMvigor210 cohort. **(E)** Scatter plot shows the correlation between B7-H3 and immune checkpoint. *P < 0.05, ***P < 0.001, & ****P < 0.0001.

We then used TIDE method to assess the potential clinical efficacy and response of immunotherapy in different B7-H3 subgroups. The high B7-H3 expression is linked to a low immunotherapy rate indicating resistance to immunotherapy in HNSCC in our findings (**Figure 4A**). Besides, we also predict the response to chemotherapy using data from the GDSC database. As depicted in **Figure 4B**, patients with the elevated expression of B7-H3 are correlated to a low IC50 score of docetaxel, cisplatin, and doxorubicin indicating a potential clinical benefit from chemotherapy.

In the IMvigor210 cohort, a similar result was found among the patients receiving anti-PD-L1 immunotherapy. High B7-H3 expression is detected in the no-response group (**Figure 4C**). Z-scores revealed that B7-H3 expression was differentially enriched between response and no response group, and most patients with no response had positive z-scores of B7-H3 (**Figure 4D**). Besides, we also analyzed the correlation between B7-H3 and immune checkpoint (SIGLEC15, HAVCR2, CD274, CTLA4, PDCD1, LAG3, and TIGIT). B7-H3 showed a positive correlation with SIGLEC15, HAVCR2 and CD274 (**Figure 4E**). These results indicate that B7-H3 has a synergistic effect with immune checkpoint in promoting the immunosuppressive microenvironment.

## Discussion

We identified B7-H3 as a novel biomarker that predicted the prognosis and response to immunotherapy in HNSCC. High B7-H3 expression was detected in HNSCC compared to a normal sample and was associated with a dismal prognosis and undesired efficacy of immunotherapy. Besides, high expression of B7-H3 was strongly related to resting CD4 T cells and macrophages M2 cells while negatively with CD8 T cell infiltration, which indicated an immunosuppressive microenvironment. Moreover, scRNA-seq analysis showed that B7-H3 was mainly expressed in malignant and stromal cells.

The B7 family comprises of inhibiting as well as activating co-stimulatory molecules that modulate immune responses negatively and positively. B7-H3, one of the ligand family members of the B7, offers a promising antibody-based immunotherapy strategy given its tumor dysregulation and significant role in tumor immunity. Earlier research has shown that B7-H3 is overexpressed and is linked with tumor progression across many kinds of human cancer (25, 26). In clear cell renal carcinoma, for example, B7-H3 is elevated and is related to the patient’s tumor node metastasis stage (27). In colorectal carcinoma, B7-H3 expression is negatively related to the OS rate (28). In line with this evidence, B7-H3 was overexpressed in HNSCC as revealed in our findings and presented excellent diagnostic value in distinguishing tumors from their matched normal tissues as evidenced by the AUC curve.

The involvement of B7-H3 in antitumor immunity has remained contentious. B7-H3 has been known as a co-stimulatory molecule for IFN-γ production and T cell activation. In the occurrence of anti-CD3 antibody imitating the TCR signal, human fusion protein B7-H3-Ig was demonstrated to improve CD4+ as well as CD8+ T cells proliferation, and the CTL activities *in vitro* are enhanced (11). However, the opposite phenomenon is also reported as evidenced by B7-H3’s capability to suppress the proliferation of T cells (12). Herein, our study revealed that in the modulation of immune cell responses in HNSCC, B7-H3 showed a negative co-stimulatory signal. The immunosuppressive cell proportion in patients with elevated expression was considerably higher, while the activated immune cell proportion was substantially lower.

In addition to their immunosuppressive functions, B7-H3 can also promote migration, angiogenesis, chemoresistance, and epithelial-to-mesenchymal transition. *In vitro* analyses have shown a substantial reduction in cancer cell adhesion to fibronectin, and a significant decrease in migration as well as invasion in B7-H3 depletion cells (29). The vital involvement of B7-H3 in cancer cell metastasis has also been highlighted in recent studies (30). Apart from cancer metastasis, B7-H3 function in angiogenesis is quickly gaining attention (31, 32). Multiple studies have demonstrated an enhanced B7-H3 expression in the endothelium of the colon, breast cancers, as well as lung (33, 34). Moreover, B7-H3 also increases the activity of the NF-κB pathway, which results in a significant increase in VEGF and IL-8 expression (31). In consistence with previous publications, our study revealed that elevated B7-H3 levels were linked to angiogenesis as well as the epithelial-mesenchymal transition pathway. Single-cell transcriptome analysis exhibited that B7-H3 is enhanced in fibroblast, endothelial, and malignant cells.

In conclusion, our results confirm the prognostic value of B7-H3 in HNSCC. We have noticed that B7-H3 expressed by the stromal as well as malignant cells may contribute to the immunosuppressive microenvironment, which presents a promising antibody-based immunotherapy strategy.

## Author’s Note

The results published here are in part based upon data generated bythe TCGA Research Network (https://www.cancer.gov/tcga).

## Data Availability Statement

The original contributions presented in the study are included in the article/supplementary material. Further inquiries can be directed to the corresponding authors.

## Author Contributions

WL and YX, conceptualization, methodology, and writing. JG and HZ, investigation. YS, software. XQ, validation. QH, formal analysis. LK and JL, project administration and funding acquisition. All authors contributed to the article and approved the submitted version.

## Funding

This work was supported by Science and Technology Commission of Shanghai Municipality (Project No. 19JC1414800).

## Conflict of Interest

The authors declare that the research was conducted in the absence of any commercial or financial relationships that could be construed as a potential conflict of interest.

## Publisher’s Note

All claims expressed in this article are solely those of the authors and do not necessarily represent those of their affiliated organizations, or those of the publisher, the editors and the reviewers. Any product that may be evaluated in this article, or claim that may be made by its manufacturer, is not guaranteed or endorsed by the publisher.
